# Quantification of santonin in eight species of *Artemisia* from Kazakhstan by means of HPLC-UV: Method development and validation

**DOI:** 10.1371/journal.pone.0173714

**Published:** 2017-03-16

**Authors:** Zuriyadda Sakipova, Nikki Siu Hai Wong, Tolkyn Bekezhanova, Sadykova, Alma Shukirbekova, Fabio Boylan

**Affiliations:** 1 Kazakh National Medical University, Almaty, Republic of Kazakhstan; 2 School of Pharmacy and Pharmaceutical Sciences, Trinity Biomedical Sciences Institute, Trinity College Dublin, Dublin, Ireland; Tallinn University of Technology, ESTONIA

## Abstract

Santonin, a powerful anthelmintic drug that was formely used to treat worms, is *Artemisia cina*'s main constituent. However, due to its toxicity to humans, it is no longer in use. Kazakhstan is looking to introduce this plant as an anthelmintic drug for veterinary purposes, despite the known toxic properties of the santonin. The objective of this study was to develop a fast and specific method for the identification of santonin and its precise quantitation using HPLC-UV in order to avoid unnecessary intoxication, which is paramount for the development of veterinary medicines. The results obtained showed that santonin appears at around 5.7 minutes in this very reliable HPLC method. The validation of the method was performed by the investigation of parameters such as precision, accuracy, reproducibility and recovery. The method was used to identify and quantify santonin in leaves of *A*. *scoparia*, *A*. *foetida*, *A*. *gmelinni*, *A*. *schrenkiana*, *A*. *frigida*, *A*. *sublesingiana*, *A terra-albae*, and *A*. *absinthium* from Kazakhstan as well as in three different extracts of leaves of *A*. *cina*. This study has provided a faster and simpler method for the identification and quantification of this compound in other species of *Artemisia* of economic importance.

## Introduction

*Artemisia cina* is a very important plant as it has high economic significance in medicines, fodder, food, ornamentals or is also used as soil fertilizers in habitats that have been disturbed. It is an endemic plant of South Kazakhstan. It is a grey-green shrub with numerous woody stems at the base, with thrice pinnately dissected leaves and small erect flowers from the tubular yellow flowers, which are usually collected when in bloom [1].

In Russian folk medicine, the water extract of *A*. *cina* was used in the treatment of bronchial asthma [2] and the alcohol extracts have larvicidal [3] and antituberculosis activity [4]. *A*. *cina*, as well as many other species of this genus, were also widely used traditionally to treat round worm infections. This activity was due to the presence of the sesquiterpene lactone santonin [5, 6]. This is the most common and most extensively studied compound from this species as well as its major compound. Santonin is the compound responsible for the anthelmintic activity of the plant and it was used for many years as a medicine to treat parasitologic diseases.

However, it is no longer in use due to its potential toxicity [5, 6, 7, 8]. Santonin is known to be toxic at the doses of 60mg for children and 200mg for adults [9]. A well-known side effect of excessive santonin intake is xanthopsia, a visual distortion where bright objects are perceived as yellow, and often dark surfaces have a violet appearance and in some serious cases, blindness can result [10, 11]. The toxicity of this compound does not occur only from overdosing, but also when it has been administered over an extended length of time as the elimination of santonin from the body happens slowly, therefore acting as a cumulative poison [6].

Kazakhstan is looking to introduce *A*.*cina* as a drug for its anthelmintic properties for veterinary purposes, despite its potential toxicity to humans [5, 6, 7, 8]. Based on that, the objective of this study was to produce an analytical tool to quantify this molecule and therefore allow for the development of a modern monograph to be included in the pharmacopoeia of Kazakhstan. A fast and specific identification of the santonin and its precise quantitation to avoid unnecessary intoxication is paramount for the development of veterinary medicines. In Kazakhstan there has been an increasing demand for herbal medicines in their National Pharmacopoeia, therefore the urgent development of modern monographs is required to be done for their endemic plants.

## Material and methods

### Reagents and materials

The solvents hexane, dichloromethane, ethanol, ethyl acetate and butanol were purchased from Trinity College Dublin HMF facilities. HPLC grade acetonitrile and chloroform were purchased from Sigma Aldrich. HPLC grade water was obtained from a deionized water treatment system from PureLab Option. The HPLC system (Waters) was used in conjunction with Waters 1525 binary HPLC pump, Waters 2487 dual λ absorbance detector, Waters 717 plus auto sampler and breeze software program. A reverse phase Thermo C18 column (250 x 4.6mm, 5μm) was used.

### Plant material

*A*. *cina* was collected on August 15th 2015 in the village Dermene, in the South-Kazakhstan region. *A*. *cina* was first dried, ground to a powder and then extracted on a UUPE 5L subcritical CO2 (SC-CO2) extractor at Phyto-aroma, Kazakhstan. The extraction time was around 18 hours with a pressure of 66- 68atm and temperature varied from 16–18°C, yielding 2.6% of extract. Almost 7g of this extract were then partitioned between hexane and methanol.

Seven other species were collected in East-Kazakhstan region in the village of Makanchi, *Artemisia gmelinii*, *Artemisia schrenkiana*, *Artemisia frigida*, *Artemisia sublesingiana*, *Artemisia terrae-albae*, *Artemisia absinthium* and *Artemisia scoparia*. Dr. Zuriyadda Sakipova was responsible for the collection of all plants used in this study. No specific permission was required for collection of them in these locations because it did not involve endangered species or protected areas. Dr. Michael Petrov from the Institute of Botany (Botanic Garden of Almaty) was responsible for the identification of all collected plant material.

### Isolation and purification of santonin

The methanol-soluble fraction (4.84g), which was separated from the subcritical CO2 extract, was submitted to successive chromatographic procedures using a chromatotron (model 7294; Harrison Research) with a 6mm silica gel GF rotor (Analtech) equipped with an FMI Lab pump. After the first run on the Chromatotron, there were large quantities of santonin contained in the fractions as a white precipitate and 1.3g of this was obtained.

The identification and elucidation of this compound was achieved using NMR spectroscopy. 1H NMR, 13C NMR, DEPT-135, DEPT-90, H-H COSY, HMBC and HSQC were recorded on the Agilent Technologies 400 MR using standard software programs and deuterated chloroform as a solvent. The structure for santonin was confirmed using x-ray crystallography. The X-ray intensity data were measured at 100(2) K using an Oxford Cryosystems Cobra low temperature device using a MiTeGen micromount. Bruker APEX software was used to correct for Lorentz and polarization effects. The data obtained for santonin was also compared with published literature data [12] for further confirmation.

**Fig 1:**
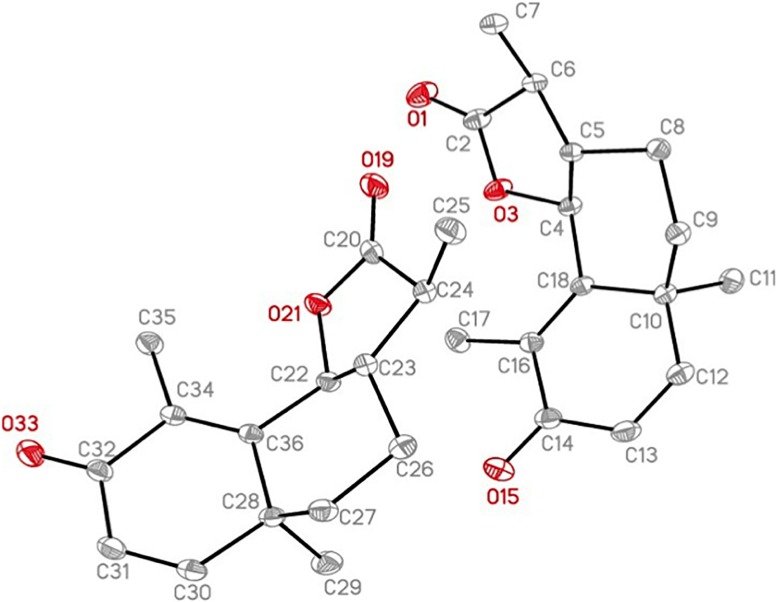
Ortep representation of the X-ray Crystal structure of α-santonin.

α-santonin, compound 2, Clear crystal; Rf 0.91 (dichloromethane: methanol; 9:1); UV λmax: 236; ESI- MS for C15H18O3 m/z; 246.29 [M+H]+; 1H-NMR (CDCl3, 400MHz) δH: 1.01 (3H, *d*, Me); 1.11(3H, *s*, Me); 1.85(3H, *d*, Me); 1.63 (2H, *m*, H-9); 1.82 (2H, *m*, H-8); 2.29 (1H, *m*, H-11); 4.66 (1H, *d*, H-6); 5.12 (1H, *s*, H-1); 5.97 (1H, *s*, H-2); 6.55 (1H, *d*, H-7); δC (150MHz, CDCl3): 10.63 (C-15); 12.24 (C-13); 22.63 (C-8); 24.78 (C-14); 37.6 (C-9); 40.59 (C-11); 41.38 (C-10); 53.47 (C-7); 81.2 (C-6); 125.26 (C-2); 127.88 (C-4); 151.83 (C-5); 155.53 (C-1); 177.78 (C-12); 186.3 (C-3). These data were in agreement to that reported by [13]. The alpha structure of santonin was confirmed using x-ray crystallography as seen in Fig 1 with an accompanying crystal data table (Table 1).

**Table 1:** Crystal data and structure refinement for santonin.

Identification code	Santonin	
Empirical formula	C_15_H_18_O_3_	
Formula weight	246.29	
Temperature	100(2) K	
Wavelength	0.71073 Å	
Crystal system	Orthorhombic	
Space group	P2_I_2_I_2_I_	
Unit cell dimensions	a = 6.9072(2) Å	α = 90°.
	b = 10.5202(3) Å	β = 90°.
	c = 34.5294(10) Å	γ = 90°.
Volume	2509.08(13) Å3	
Z	8	
Density (calculated)	1.304 Mg/m3	
Absorption coefficient	0.090 mm-1	
F(000)	1056	
Crystal size	0.270 x 0.230 x 0.230 mm3	
Theta range for data collection	2.024 to 30.596°	
Index ranges	-9≤h≤9, -15≤h≤15, -49≤h≤49	
Reflections collected	84198	
Independent reflections	7686 [R(int) = 0.0210]	
Completeness to theta = 25.242o	99.90%	
Absorption correction	Semi-empirical from equivalents	
Max. and min. transmissions	0.7461 and 0.7021	
Refinement method	Full-matrix least-squares on F2	
Data / restraints / parameters	7686 / 0 / 331	
Goodness-of-fit on F2	1.021	
Final R indices [I>2τ(I)]	R1 = 0.0282, wR2 = 0.0796	
R indices (all data)	R1 = 0.0292, wR2 = 0.0805	
Absolute structure parameter	-0.06(9)	
Largest diff. peak and hole	0.295 and -0.168 e.Å-3	

### Sample preparation

A total of 200g of *A*. *cin*a leaves were re-collected and dried. The leaf compounds were extracted using supercritical CO2 with conditions set at 350atm, 60oC for two hours. The final yield was 40g of extract.

Also, 150g of dried *A*. *cina* were extracted with chloroform at 45 degrees Celcius to obtain a total of 4g of extract. So in this way, three main extracts were prepared: subcritical CO2, supercritical CO2 and chloroform.

The leaves of the seven other *Artemisia* species were dried and ground into powder and 1g of each were taken and soaked in 25ml of chloroform and 25ml of methanol. After extraction for three hours on a stirring plate, the extracts were filtered and dried using a rotary evaporator (model: IKA HB10 control attached to a Fisher Scientific pump). Samples were re-suspended in 10 ml of methanol prior to be analysed by HPLC.

### Preparation of standard solutions

#### Reference solution

About 1mg of santonin was accurately weighed out in a glass vial and dissolved in 1ml of acetonitrile.

#### Test solution

About 10mg of the supercritical CO2 extract was weighed out in a glass vial and dissolved in 5ml of chloroform and 5ml of acetonitrile. The vial was then placed in a water bath at 60oC to ensure that everything was dissolved to avoid particles present to block the column and cause the machine to clog. To ensure that there were no particles suspended in the solution, each sample was filtrated using a 0.45mm filter (Nylon66) before placement into the HPLC machine.

### UV-HPLC analysis

Ten microlitres of the samples and standard solutions were injected in quadruplicates and eluted using the following gradient at a flow of 1.0ml/min with water (A) and acetonitrile (B): 0min, 35:65 (A:B); 5min, 35:65; 10min, 45:55; 15min, 55:45; 20min, 65:45. According to [12], the machine was set to detect peaks at 236nm and the pressure was at a maximum of 5000atm.

### Method validation

#### Calibration curve

Reference solutions were prepared by diluting santonin with acetonitrile in the following concentrations: 1250μg/ml; 625μg/ml; 312.5μg/ml; 156.25μg/ml; 78.13μg/ml; 39.06μg/ml; 19.53μg/ml; 9.77μg/ml; 4.88μg/ml. These solutions were analysed in quadruplicates (n = 4) using the conditions already described. These results allowed for a calibration curve to be constructed. The Student’s *t*-test was used to investigate the intercept and the regression coefficient. Both the calibration line and the residuals were clearly examined and assessed.

#### Precision

Eleven replicate samples were made fresh for each day using the methods mentioned above and these were analysed on three separate days on the same equipment, by the same analyst to ensure that the technique could be replicated and also to reduce the chances of errors made if the experiment was carried out by multiple people.

#### Accuracy

The recovery experiment was carried out to investigate the accuracy of this method for santonin. The samples were prepared using concentrations of santonin at 1250μg/ml, 625μg/ml and 312.5μg/ml and each of these were mixed with 1mg/ml of the CO2 extract solution. The ratio of the samples prepared in was 1:1 santonin and CO2 extract, respectively. These solutions were injected into the HPLC machine and the recovery was calculated by comparing what the theoretical value of santonin should be and what the value actually was from the experiment, expressed in percentage.

#### Application of method

Each sample of the different species was investigated against santonin using the same method devised in this study.

## Results

### Development of the chromatographic method

**Fig 2:**
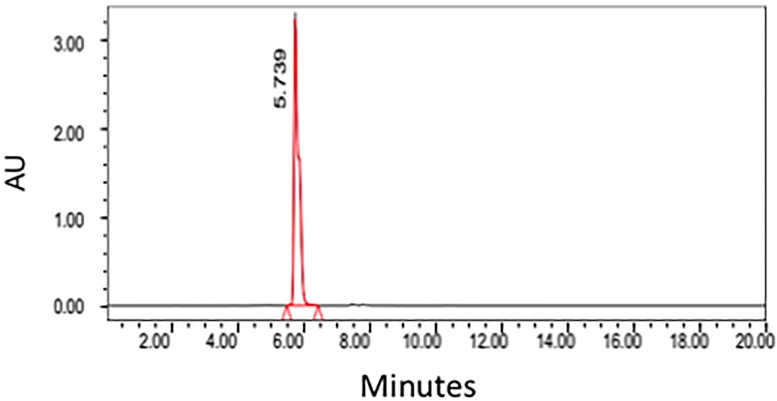
HPLC Chromatogram of santonin obtained from *Artemisia cina* Berg.

**Fig 3:**
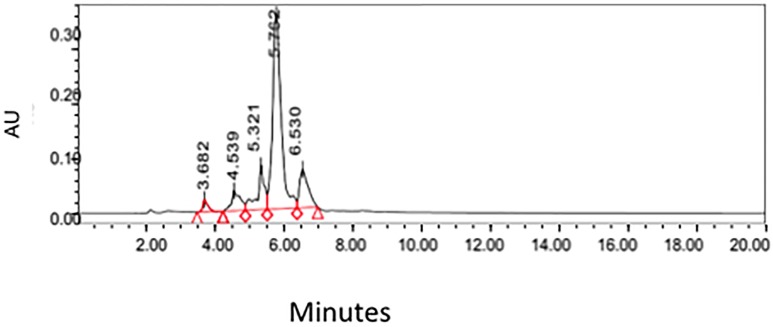
HPLC Chromatogram obtained from the supercritical CO_2_ extract of *Artemisia cina* Berg.

A HPLC method for the analysis of santonin was devised using parameters interpreted from previous publications [5, 14]. The standard of santonin was run in the HPLC machine using a C18 column showing a retention time of approximately 5.7 minutes (Fig 2). The supercritical CO2 crude extract was ran under the same conditions as the standard and clearly showing the santonin peaking at roughly the same time, 5.7 minutes (Fig 3).

### Method validation

#### Accuracy

**Fig 4:**
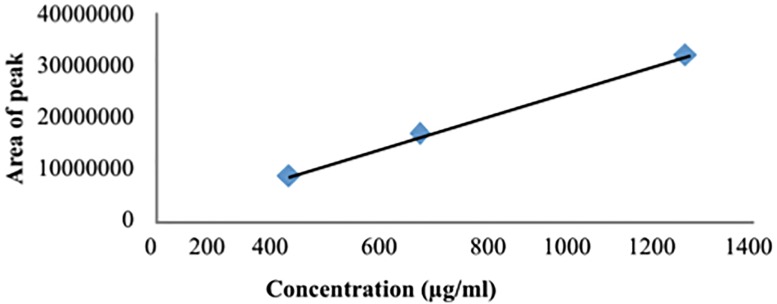
The trend line of the three concentrations used for the accuracy test with R^2^ = 0.99.

A calibration curve was made (Fig 4) using the average area under the curve (n = 4) of the three concentrations of the santonin (312.5, 625 and 1250μg/ml) The equation of the line was used to calculate the theoretical area under the curve and this was compared with the actual area under the curve to examine the accuracy of the method for quantification of santonin (Table 1).

Table 2 clearly shows the accuracy of this method demonstrating that over 95% of the santonin was recovered from the supercritical CO2 extract and over 80% from the chloroform extract using this method.

**Table 2:** The results of the accuracy test.

Concentration of Santonin μg/ml	Actual Area	Theoretical Area	Percentage (%)
Supercritical CO_2_			
1250	18997515.7	19002023	99.98
625	10924186.3	11386015	95.94
312.5	7061784.1	7300633	96.73
Chloroform			
1250	13995363.7	17067171.5	82.00
625	8593971.9	9451163.5	90.93
312.5	5346506.3	5365781	99.64

#### Precision

Calibration curves for santonin at 9 different concentrations (1250μg/ml; 625μg/ml; 312μg/ml; 156.25μg/ml; 78.13μg/ml; 39.06μg/ml; 19.53μg/ml; 9.77μg/ml; 4.88μg/ml) in replicates of four (n = 4) were run in four different days. In all graphics, the results were very similar and the R2 value obtained was always above 0.99 indicating that the results were significant. The calibrations curves were used for the quantification of santonin later in the studied samples.

#### Quantification

**Fig 5:**
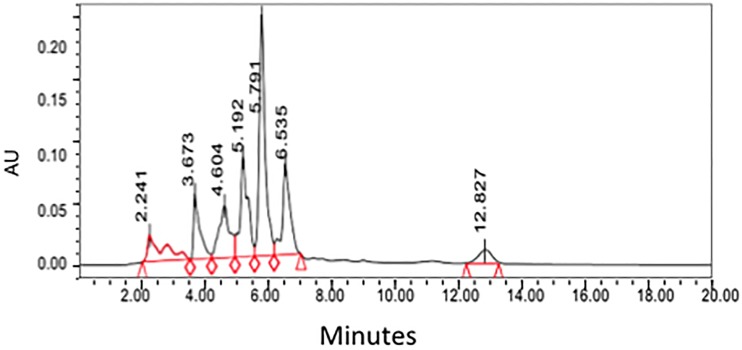
The HPLC of the chloroform extract from *Artemisia cina*.

**Fig 6:**
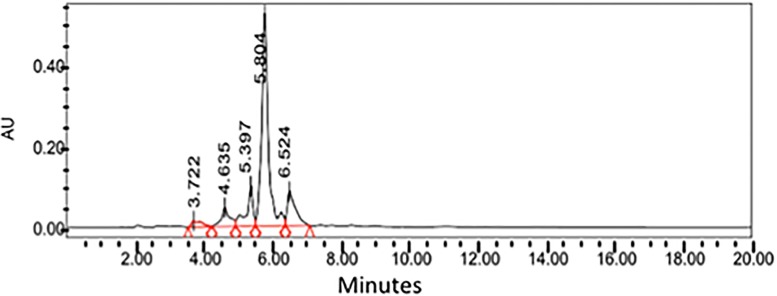
The HPLC of the supercritical CO_2_ extract from *Artemisia cina*.

Figs 5 and 6 show the HPLC separation of the chloroform and supercritical extract, respectively using the method devised from this study. Chloroform extraction seems to extract more compounds overall when compared to the supercritical CO2 extraction method.

Upon investigating the quantity of santonin at approximately 5.7 minutes in the HPLC of these extracts,there were significant differences in the quantity of santonin extracted using the two different techniques with the santonin quantity in the supercritical CO2 being 3 times higher than that of the santonin from the chloroform extract (Table 3). The concentration of santonin found in the extracts fell within the concentrations used to elaborate the standard curve.

**Table 3:** Concentration of santonin in chloroform and supercritical CO_2_ extracts.

Extract	Concentration value for santonin (μg/ml)				Average (μg/ml)	Average (g/100g dry leaf)
Chloroform	86.32732	85.66273	80.89646	80.45068	83.3343	0.83
Supercritical CO_2_	254.2992	250.6032	248.6882	248.0381	250.4072	2.5

## Discussion

As it is known that the major compound santonin has potential toxicity [5, 6, 7, 8] it is of interest to be able to quantify this compound in plants from the *Artemisia* genus or from any other plants that could contain santonin. The development of this HPLC method was based on previous papers and studies on santonin and sesquiterpene lactones as there has been little research carried out with *A*. *cina*. Miraldi *et al*. (1998) devised a new method to extract and quantify santonin using High Performance Liquid Chromatography in *Artemisia caerulescens* ssp. *cretacea* (Fiori) Br.-Catt. & Gubell.

In their experiment Miraldi *et al*. made modifications to previous methods in an attempt to devise a faster, easier and more accurate extraction and HPLC analytical procedure. The mobile phase consisted of an isocratic elution with acetonitrile: water (4: 6) for the duration of 10 minutes (a flow rate from 0.5 mL/min to 0.8 mL/min). This was followed by a 5 minute linear gradient of acetonitrile: water from 4:6 to 6:4 with a flow rate from 0.8 mL/min to 1.0 mL/min. and then back to an isocratic elution with acetonitrile: water (6:4) for an additional 15 minutes (flow rate 1.0 mL/min). The UV absorbance was monitored at 236nm. The injection volume was 20 μL in all the experiments and all their analyses were conducted at room temperature. Their calibration curve was made by using the injections of five different concentrations of santonin varying from 0.00625g to 0.1 g. At each concentration, the values obtained were acceptable (absolute error 0.10 min; relative error 5.0%).

Their results showed santonin to have a retention time of approximately 14 minutes. By using the method devised in this study, the retention time for santonin fell to less than 6 minutes, more than 8 minutes faster than the method devised by Miraldi *et al*. (1998).

Upon investigation the accuracy and precision of the method devised in this paper, the recovery of santonin from the supercritical CO2 extract was over 95% while its recovery from the Chloroform extract was over 90%. This confirms the validity of this method, which can also be used to examine santonin presence in other species.

To finalize our validation, seven other *Artemisia* species were quantified for santonin using this method. The calibration curve previously obtained shows a valid regression line y = 25705x - 1540 (R^2^ = 0.99) which was used for the quantification of the plant species listed in Table 3. Preparation of the plant extracts is described in material and methods. Ten microliters of each extract was injected in triplicate.

It is clearly shown in Table 4 that *Artemisia cina* possess the most santonin (1.96g/100g leaves) and several other species show no santonin present. Most of the other species with close to zero or zero santonin content were traditionally used as oral medications in some countries and are still in practise now. *A*. *gmelinii* leaves were used in south and south-east Asia to treat inflammatory liver conditions [15]. *A*. *absinthium* was used by the indigenous as an antispasmodic, for the restoration of declining mental function, febrifuge, anthelmintic and inflammation of the liver, and to improve memory [16]. *A*. *scoparia* is well known in Pakistan to treat pain, inflammation and febrile conditions [17]. In Mongolia, *A*. *frigida* is commonly used as in folk medicine to treat joint swelling, sore carbuncle, and abnormal menstruation [18]. It is important to be able to quantify santonin in these plants seasonally.

**Table 4:** The quantification of santonin in seven *Artemisia* species using HPLC.

Plant Species	Concentration of Santonin (g/100g plant material)
*Artemisia cina*	1.96
*Artemisia gmelinii*	0
*Artemisia schrenkiana*	0
*Artemisia frigida*	0.22
*Artemisia sublesingiana*	0.16
*Artemisia terrae-albae*	0
*Artemisia absinthium*	0
*Artemisia scoparia*	0.04

It was also of interest to see if there were any differences in the quantity of santonin isolated using different extraction techniques. This is because techniques such as supercritical and subcritical CO_2_ extractions are more expensive than those using solvents such as chloroform or ethanol. In this case, as seen in Table 3 the average concentration of santonin extracted using chloroform was 83.33μg/ml whereas the concentration of santonin extracted using supercritical CO_2_ was 250.40μg/ml. These results report a clear difference in the quantity of isolated santonin using the different methods of extraction. They also provide useful information for selecting extraction techniques based on the quantity of santonin allowed in the extract for medicinal purposes (veterinary medicine).

However many difficulties are present when attempting to quantify compounds in a plant because their contents are affected by many variables. For example, it is common to observe diurnal variations in plant secondary chemical components [19] as well as climatic, physiological and environmental variations [20, 21]. This may be a problem if quantification of compounds is required with many replications and thus needing numerous amounts of plant samples. If the collection of these plants is not standardized then the results obtained may not give a true value or the desired outcome. In the case of santonin, the content is at a maximum when the flowering dried tops of the plants are collected at full bloom [5], also an important factor to be considered when preparing any effective veterinary medicine with minimum toxic effects.

The potential for *A*. *cina* to be reintroduced in Kazakhstan as a veterinary medicine is dependent on the quantity of santonin in the plant to have effect but with minimum toxic activity. This registration of veterinary medicines does not require as much specialized expertise and examinations compared to human medicines. Although the toxicity for santonin at the doses of 60mg for children and 200mg for adults is well documented, we also know that the LD50 for mammals is in the range of 900mg/kg [22], which can still potentially allow the use of this plant in veterinary medicine. The dosage for animals will also be much less than that given to humans and moreover, it may be possible, with chemical modification, to keep or improve the activity and decrease the toxicity of *A*. *cina*.
