# Correction: Quantification of santonin in eight species of *Artemisia* from Kazakhstan by means of HPLC-UV: Method development and validation

**DOI:** 10.1371/journal.pone.0175088

**Published:** 2017-03-28

**Authors:** The PLOS ONE Staff

The fourth author’s name appears incorrectly. The correct name is: Aida Sadykova. The correct citation is: Sakipova Z, Wong NSH, Bekezhanova T, Sadykova A, Shukirbekova A, Boylan F (2017) Quantification of santonin in eight species of *Artemisia* from Kazakhstan by means of HPLC-UV: Method development and validation. PLoS ONE 12(3): e0173714. doi:10.1371/journal.pone.0173714. The publisher apologizes for the error.

## References

[1] Sakipova Z, Wong NSH, Bekezhanova T, Sadykova, Shukirbekova A, Boylan F (2017) Quantification of santonin in eight species of *Artemisia* from Kazakhstan by means of HPLC-UV: Method development and validation. PLoS ONE 12(3): e0173714 doi:10.1371/journal.pone.0173714 28301522 10.1371/journal.pone.0173714 PMC5354383

